# Immune-driven alterations in mucin sulphation is an important mediator of *Trichuris muris* helminth expulsion

**DOI:** 10.1371/journal.ppat.1006218

**Published:** 2017-02-13

**Authors:** Sumaira Z. Hasnain, Paul A. Dawson, Rohan Lourie, Peter Hutson, Hui Tong, Richard K. Grencis, Michael A. McGuckin, David J. Thornton

**Affiliations:** 1 Inflammatory Disease Biology and Therapeutics Group, Mater Research Institute - The University of Queensland, Translational Research Institute, Brisbane, Australia; 2 Mater Pathology Services, Mater Hospitals, South Brisbane, Queensland, Australia; 3 Manchester Immunology Group Medicine and Health, Manchester Academic Health Sciences Centre, University of Manchester, Manchester, United Kingdom; 4 Wellcome Trust Centre for Cell-Matrix Research, Faculty of Biology, Medicine and Health, Manchester Academic Health Sciences Centre, University of Manchester, Manchester, United Kingdom; Uniformed Services University of the Health Sciences, UNITED STATES

## Abstract

Mucins are heavily glycosylated proteins that give mucus its gel-like properties. Moreover, the glycans decorating the mucin protein core can alter the protective properties of the mucus barrier. To investigate whether these alterations could be parasite-induced we utilized the *Trichuris muris* (*T*. *muris*) infection model, using different infection doses and strains of mice that are resistant (high dose infection in BALB/c and C57BL6 mice) or susceptible (high dose infection in AKR and low dose infection in BALB/c mice) to chronic infection by *T*. *muris*. During chronicity, within the immediate vicinity of the *T*. *muris* helminth the goblet cell thecae contained mainly sialylated mucins. In contrast, the goblet cells within the epithelial crypts in the resistant models contained mainly sulphated mucins. Maintained mucin sulphation was promoted by T_H_2-immune responses, in particular IL-13, and contributed to the protective properties of the mucus layer, making it less vulnerable to degradation by *T*. *muris* excretory secretory products. Mucin sulphation was markedly reduced in the caecal goblet cells in the sulphate anion transporter-1 (Sat-1) deficient mice. We found that Sat-1 deficient mice were susceptible to chronic infection despite a strong T_H_2-immune response. Lower sulphation levels lead to decreased efficiency of establishment of *T*. *muris* infection, independent of egg hatching. This study highlights the complex process by which immune-regulated alterations in mucin glycosylation occur following *T*. *muris* infection, which contributes to clearance of parasitic infection.

## Author summary

Approximately 2 billion people are infected with worms every year, causing physical, nutritional and cognitive impairment particularly in children. Mucins are large sugar-coated (glycosylated) proteins that form the intestinal mucus layer. This mucus layer protects our ‘insides’ from external insults and plays an important role during worm infection. We discovered that there is a difference in the glycosylation of mucins in people infected with worms compared to uninfected individuals. Therefore, using different mouse models we investigated the role of glycosylation, and in particular sulphation of mucins in infection. We found that mucin glycosylation is controlled by the immune response and increased sulphation correlated with the expulsion of the worm from the host. Highly sulphated mucins were protected from degradation by the worm. Moreover, mice lacking a sulphate transporter had significantly lower sulphation levels on mucins, which resulted in a reduction in the establishment of the worms and chronic infection.

## Introduction

The intestinal epithelium is lined by a continuous mucus barrier which provides physical protection and chemically protects the epithelial cell layer by sequestering important host defence factors within its complex matrix [1]. Gel-forming mucins (Muc2 in the intestine) produced by goblet cells, give mucus its gel-like properties and play a significant role in protection against helminth infections [2]. Mucins are large heavily glycosylated proteins, predominantly consisting of O-glycans which account for at least 70% of their molecular weight [3]. The O-linked sugars are assembled progressively by glycosyltransferases on to a serine or threonine residue, found in the serine-threonine-proline rich tandem repeat regions of the mucin protein core [1]. These glycan chains have well-established fundamental roles in many biological processes including in inflammatory responses [4].

Changes in mucin glycosylation have been previously described in murine parasitic infections. Whether these changes are important in the protective function of the mucus barrier, however, has not yet been established. Several studies have investigated the direct role of glycans as protective elements that attach and clear pathogens from the gut [5–8], but most of the evidence for the protective role of the glycans present on mucins comes from animal models. A change in the glycans can lead to inflammation because of the alteration in the protective properties of the mucus barrier [9–12]. For example, the terminal sugar fucose has been shown to be crucial in host-pathogen interaction in *Helicobacter pylori* infection [13]. Moreover, inducing colitis in mice deficient in the N-acetylglucosamine-6-O-sulphotransferase-2 [14] which is expressed highly in the colon, results in a significantly higher leukocyte infiltration and is thought to exacerbate inflammation. Mice lacking either core 1- or core 3-derived O-glycan chains, or both, develop spontaneous colitis with the double knockout mice having the most severe and widespread disease [15–17].

Previous studies have shown that inducing mucin sulphation with reserpine *in vivo* reduces the establishment of infection with the intestinal helminth *Strongyloides venezuelensis* [18]. Sulphotransferases were also up-regulated shortly before the rejection of the helminth, *Nippostrongylus brasiliensis* and have been suggested to play a protective role during worm expulsion [19,20]. In addition, the up-regulation of sialomucins observed in other helminth models such as *Trichinella spiralis*, which have been found to be regulated by T cells, are thought to be protective [9,10]. Moreover, gastrointestinal disorders such as ulcerative colitis and Crohn’s disease are associated with a loss of mucin sulphation [21,22]. Whether the changes observed in mucin glycosylation in human colitis occur as a consequence of disease or are an active alteration important in resolving the disease has yet to be fully elucidated. Mucin glycosylation is known to be important in maintaining intestinal homeostasis and an absence of mucin glycosylation results in inflammation [17,23]. It was shown that a reduction in mucin sulphation, in particular, can lead to an increase in susceptibility to colitis and bacterial infections [24]. In the intestine, a rich commensal flora is maintained within the outer mucus layer and it is thought that the microbiota can influence the level of sulphation in the intestine, which could in turn effect mucin sulphation [25]. Paradoxically, mucin sulphation can also protect the mucin core from bacterial glycosidases [4]. Overall, it is thought that the alterations in glycosylation can lead to reduced effectiveness of the mucus barrier, which in turn may exacerbate inflammation.

The intestinal niche occupied by nematodes, in particular the caecum, contains mucins that are more sulphated than in any other site in the body [21]. Using the *Trichuris muris* (*T*. *muris*) mouse model, we have shown that intestinal mucins play an essential role in the expulsion of this helminth from the host [2,26]. Therefore, the changes in mucin sulphation could alter the protective nature of the mucus barrier and affect helminth establishment and/or expulsion. *T*. *muris* helminth infection model provides a robust model of both acute and chronic infection. Using this model, we observed a clear switch in glycosylation from sulphomucins to sialomucins during chronic infection in the *T*. *muris* murine model. For the first time, we also demonstrate that mucin sulphation is influenced by IL-13 and mucus with high sulphomucin content is more resistant to degradation by the *T*. *muris* excretory secretory products (ESPs). Moreover, underlining the functional importance, Sulphate anion transporter1 (Sat1)-deficient mice with reduced mucin sulphation developed chronic *T*. *muris* infection. Interestingly, a preliminary histopathological examination of individuals infected with *Enterobius vermicularis*, a highly prevalent gastrointestinal dwelling nematode of human cecum showed a decreased mucin sulphation staining supportive of the mouse studies.

## Results

### Reduced sulphation of Muc2 during chronic *T*. *muris* infection

**Fig 1:**
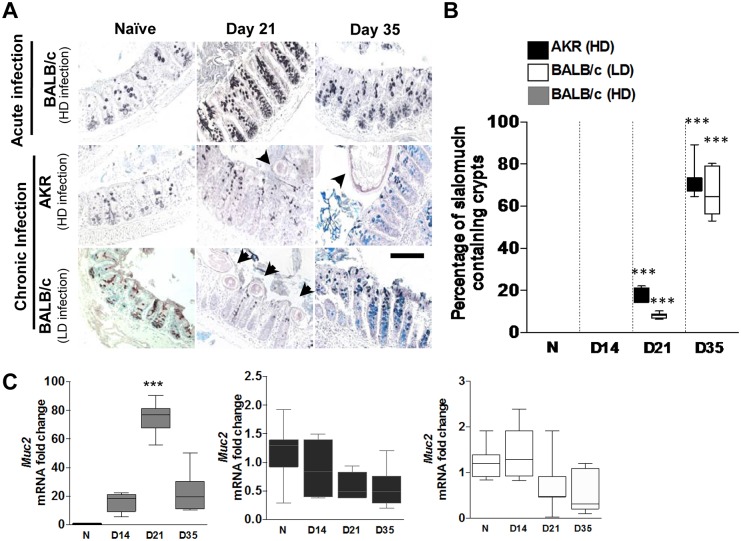
Reduced sulphation in chronic mouse helminth infection. (A) HID-AB staining of the caecum of mice with acute infection (BALB/c high dose (HD)) and mice susceptible to chronic infection (AKR HD and BALB/c low dose (LD)) were used to differentiate between sulphomucins (black staining) and sialomucins (blue staining) during *T*. *muris* infection. Helminths are highlighted by black arrows. Representative images from n = 5–6 mice per group. Scale bar = 100 μm. (B) Enumeration of the percentage of crypts containing sialomucins during the course of infection. Crypts from BALB/c mice with HD infection were occupied by sulphated mucins at all times. (C) qRT-PCR was used to determine the mRNA levels of Muc2 in BALB/c mice with high and low dose infection and AKR mice with high dose infection. Results from n = 5–6 mice, box plots show median, quartiles and, range. *** = P<0.001. One-way ANOVA with Bonferroni post-test.

To gain an understanding of whether mucin glycosylation plays an important role during helminth infection, we utilised the *T*. *muris* model of helminth infection in mice. The degree of mucin sulphation and sialylation were assessed during *T*. *muris* infection with High-Iron Diamine-Alcian Blue (HID-AB) staining in acute (high dose (HD) infection in BALB/c mice) and chronic (HD and low dose (LD) infection in AKR and BALB/c mice, respectively); worm burdens are shown in S1a Fig. Using HID-AB staining a distinction can be made between sulphomucins (black) and sialomucins (blue), present in the goblet cell thecae (Fig 1A). Normally in the caecum, the niche of the parasite, the mucins are predominantly highly sulphated with little evidence of sialomucin-containing goblet cells (Fig 1A—naïve levels). In the mice with acute infection, as infection progressed, there was goblet cell hyperplasia and the expected increase in *Muc2* expression (Fig 1C) [2]. Importantly, mucins present within the goblet cells in the caecal crypts were all majorly sulphated (Fig 1B). However, in the chronic models of infection, there was a decrease in *Muc2* expression (Fig 1C), accompanied by a loss of sulphated goblet cell thecae and an increase in sialomucins within the goblet cells. This switch in glycosylation was localised to the caecum; no major changes were observed in the colon (S1B Fig).

### Up-regulation of sulphotransferase genes in acute infection

**Fig 2:**
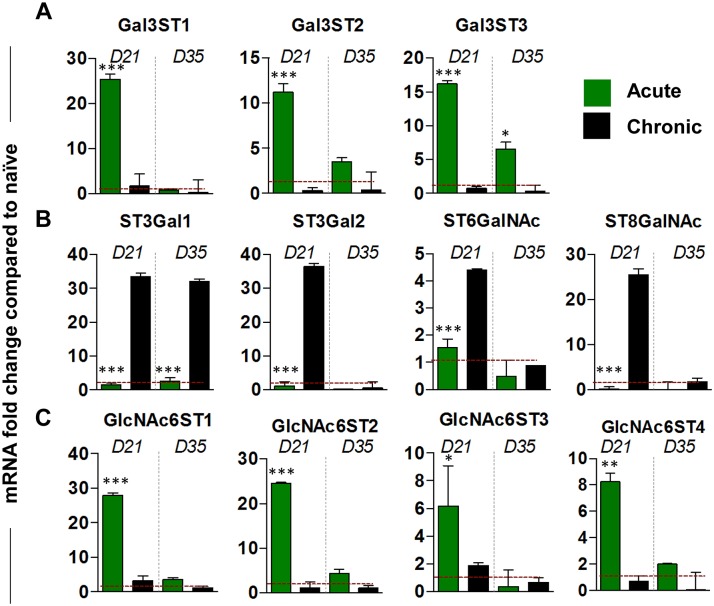
Sulphotransferases are upregulated in epithelial cells following acute infection. Caecal epithelial cells were isolated and qRT-PCR was used to determine the levels of (A) sulphotransfereases *Gal3St1*, *Gal3St2* and *Gal3St3*; (B) Sialyltransferases, *ST3Gal1*, *ST3Gal2*, *ST6GalNAc* and *ST8GalNac*, (C) glucosamine-O-sulphotransferases *GlcNAc6ST1*, *GlcNAc6ST2*, *GlcNAc6ST3* and *GlcNAc6ST4*; during acute and chronic *T*. *muris* infection. Red dashed line = naïve levels. Results are presented as mean ± SEM of 5–7 mice per group. Unpaired student t-test, *P<0.05, **P<0.01, ***P<0.001, Acute (BALB/c HD) Vs. Chronic (BALB/c LD) infection.

Mucin glycosylation is dependent on the array of glycosyltransferases present within the Golgi apparatus of the goblet cells. Therefore, considering the differences identified, the changes in the expression of major sulpho- and sialotransferases in isolated caecal epithelial cells was determined using qRT-PCR (Fig 2). Interestingly, mirroring the increase in sulphated mucins present in goblet cells, the galactose-O-sulphotransferases *Gal3St1*, *Gal3St2* and *Gal3St3* (Fig 2A) and glucosamine-O-sulphotransferases *GlcNAc6ST1*, *GlcNAc6ST2* and *GlcNAc6ST4* (Fig 2B) were highly up-regulated around the time worm expulsion is occurring on day 21 pi. in acute infection (BALB/c HD) but not during chronic infection (BALB/c LD; worm burden data is shown in S1A Fig). In acute infection, the level of sulfotransferase expression then reduced after worm clearance paralleling the result for *Muc2* expression (S1B Fig). In contrast, in chronic infection in the same strain of mice infected with a low dose of worms, the sulphotransferase genes were not induced but the sialyltransferases *ST3Gal1*, *ST3Gal2* and *ST8GalNAc* were upregulated on day 21, with ST3Gal1 remaining high at day 35 pi (Fig 2C). Thus induction of a spectrum of sulphotransferase genes occurs concomitantly with worm expulsion.

### Inducing resistance in susceptible mice reversed mucin glycosylation from sialylated to sulphated

**Fig 3:**
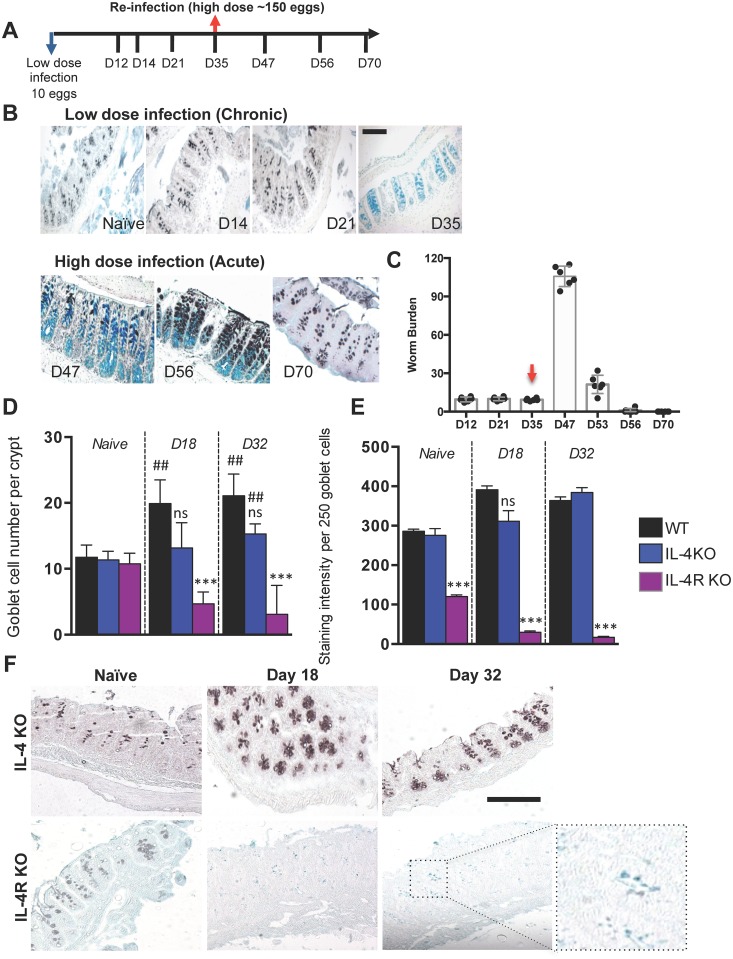
Changes in mucin glycosylation are dependent on the immune response and are reversed after worm clearance. (A) Schematic depicting the time course of infection in BALB/c mice in the low dose/high dose reinfection experiment. (B) HID-AB staining depicting the changes in sulpho- and sialomucins during the course of the infection experiment in (A). (C) Worm burdens assessed on day 12, 21, 35, 47, 53 and 56 post infection (pi); red arrow depicts the time of re-infection with a high dose of *T*. *muris* eggs. (D) Goblet cell numbers quantified in wild-type, IL-4 knockout (KO) and IL-4R KO uninfected mice and 18 and 32 days after infection with 150 *T*. *muris* eggs. (E, F) Caecal tissue from infected and non-infected IL-4 KO and IL-4R KO mice stained with HID-AB; (E) depicts the HID staining intensity measured per 250 goblet cells. Scale bar = 100 μm. Results are presented as mean ± SEM of 5–7 mice per group. One-way ANOVA with Bonferroni post-test. *P<0.05, **P<0.01, ***P<0.001 compared to WT and #P<0.05, ##P<0.01, ###P<0.001 compared to naïve controls.

We wanted to determine whether the changes in glycosylation were a direct result of infection itself and whether these changes in mucin glycosylation could be altered post infection. Therefore, to address these questions, BALB/c mice were infected with a low dose of *T*. *muris* eggs [27]; this resulted in a chronic infection, as adult worms were still present in the caeca of these mice on day 35 pi. (Fig 3A and 3C). As expected, by day 35 pi. no changes in mucin expression were observed (S2C Fig) in these mice however, goblet cells predominantly contained sialomucins (Fig 3B).

Subsequently, at day 35 pi., which is considered to be a chronic infection, these mice were re-challenged with a high dose (>150) of *T*. *muris* eggs (Fig 3A). A second infection was established in these mice, as approximately 100 worms were present in the caeca after 12 days (day 47 pi.) of re-infection (Fig 3C). However, most worms were eradicated 21 days after reinfection (day 56 pi.) and no worms were present in the caecum by 35 days post-reinfection (day 70 pi.). Re-challenging these chronically infected mice with a high dose infection resulted in the induction of *Muc2* and *Il13* expression and a reduction in *Ifnγ* expression (S2A and S2B Fig). Histological analysis of the caeca with HID-AB staining revealed as expected the change from goblet cells containing predominantly sulphomucins to sialomucins in chronic infection (Fig 3B). These changes were reversible and goblet cells changed from producing sialomucins to sulphomucins after reinfection (Fig 3B), which correlated with *T*. *muris* worm expulsion (Fig 3C).

### Sulphation of mucins is controlled by IL-13

It is well-established that the mice resistant to *T*. *muris* infection mount strong T_H_2-type immune responses and those susceptible to chronic infection exhibit T_H_1-type immune responses (as can be seen from the relative caecal expression of genes encoding the T_H_2 and T_H_1 cytokines *Il13* and *Ifnγ* in S2 Fig) [28]. To assess the role of the T_H_2-type immune response and in particular, IL-4 and IL-13, in mucin sulphation, IL-4 knockout (KO) and IL-4R KO mice on the BALB/c background were infected with a high dose of *T*. *muris* eggs. The IL-4 KO mice were able to expel worms, however worm expulsion was slightly delayed in these mice (S3A Fig), as reported previously [29]. On day 18 pi. goblet cell hyperplasia was less pronounced in the IL-4 KO mice when compared to the wild-type BALB/c mice, although this was not significantly different (Fig 3D). A significant increase in the number of goblet cells was observed in the IL-4 KO mice by day 32 post infection compared to naïve mice. Furthermore, HID-AB staining showed that goblet cells in the IL-4KO mice predominantly contained sulphated mucins (Fig 3E and 3F). As IL-4 and IL-13 act through a heterodimeric receptor involving IL-4Rα, IL-4R KO mice do not respond to either cytokine and are unable to mount strong T_H_2-type immune responses, and consequently are susceptible to chronic *T*. *muris* infection (worm burden; S3A Fig) [29]. Importantly, uninfected control IL-4R KO mice had significantly lower levels of sulphation compared to the naïve wild type and IL-4 KO mice (Fig 3D–3F). This was reflected by very substantial reduction in the sulphotransferase genes, *Gal3ST1*, *GlcNAcST2*, *GlcNAc6ST3* in the naïve IL-4R KO mice (S3B Fig), the other sulphotransferase genes shown in Fig 2 remained unaltered (S3G Fig). As infection progressed, a decrease in the number of goblet cells was also observed in the IL-4R KO mice compared to naïve and IL-4 KO mice. Moreover, the goblet cells lost their ‘goblet like morphology’, exhibiting very small thecae, which stained blue with HID-AB, indicative of predominantly containing sialylated mucins (Fig 3F).

### Sulphomucins are less prone to degradation by *T*. *muris* excretory secretory products

To determine whether the differences in sulphomucin content alters the properties of the mucus barrier, we compared mucus from wild-type mice and NaS1 KO mice, which lack the Na^+^-sulphate transport 1 (Slc13a1)[30]. Slc13a1 is primarily expressed in the ileum/caecum/colon and kidney where it mediates sulphate absorption and reabsorption, respectively. Deletion of *Slc13a1* in mice leads to hyposulfataemia and reduced sulphonation capacity which leads to depleted intestinal sulphomucin content (S4A Fig [24]). Mucus extracted from the uninfected wild-type and NaS1 mice was subjected to agarose gel electrophoresis and western blotted. Staining with HID confirmed that mucin sulphation was significantly reduced in the NaS1 mice compared to wild-type mice, with PAS staining (reacts with all mucin carbohydrates) used to confirm that comparable amounts of glycoproteins were isolated and compared (S4B Fig).

The excretory/secretory products (ESPs) released by *T*. *muris*, contains a mixture of enzymes. We have previously demonstrated that serine proteases secreted by the helminth can specifically depolymerise the Muc2 mucin polymers that give mucus its viscoelastic properties [31]. Therefore, we assessed the contribution of the mucin sulphation to the resistance to depolymerisation of Muc2. Caecal mucus was isolated from wild type and Nas1 KO mice and treated with 50 μg/ml of ESPs from *T*. *muris* for 2 or 6 hours at 37°C to determine whether ESPs can alter mucus. Treated or untreated mucus was subsequently subjected to rate zonal centrifugation to assess the change in size and/or shape of mucins by analysing their distribution by PAS-staining. The sedimentation profiles of untreated glycoproteins from wild-type (high sulphomucin content) and NaS1 KO (lower sulphomucin content) were comparable with broad peaks (fractions 9–20) (S4C Fig). After treatment with ESPs from *T*. *muris*, the sedimentation profile of glycoproteins from NaS1 KO mice was altered, whereas the profile of wild type glycoproteins was substantially unchanged. The PAS-positive material from the NaS1 KO mice shifted to the top of the gradient (fractions 4–17) compared to PAS-positive material from the wild-type mice present in fractions 9–20, consistent with reduced size of mucin polymers (S4C Fig; quantification of rate zonal data presented as percentage of area under the curve shown in S4C Fig). This experiment shows that mucus with lower sulphomucin content is more susceptible to the degradative effects exerted by the *T*. *muris* ESPs.

### Lower mucin sulphation does not affect worm establishment or expulsion in NaS1 KO mice

High sulphomucin content that accompanies helminth rejection was predominately controlled by IL-13. This was confirmed *in-vitro* by treating the intestinal cell lines, LS174T cells, with 50 ng/mL of IL-13 for 24 h. We observed an increase in mucin production, which was accompanied by an increase in sulphotransferase and a decrease in sialyltransferase genes (S3C and S3D Fig). Treatment of LS174T cells with IFN-γ lead to an increase in sialytransferases (S3E Fig), whereas no alterations in sulphotransferases were observed (S3E Fig). We also examined degradation by *T*. *muris* ESPs of mucins produced by LS174T cells with and without IL-13 stimulation and found that sulphated mucins induced by IL-13 treatment are more resistant to degradation by parasite ES products. Moreover, sialylated mucins induced by IFNγ treatment were degraded rapidly compared to control mucins (S3F Fig). As IL-13 is an essential mediator in the expulsion of helminths and altered sulphation levels via the regulation of sulphotransferases, we hypothesised that the level of mucin sulphation in the caecum could affect *T*. *muris* infection, in particular worm establishment and rejection. To address this possibility, NaS1 KO and their wild-type littermates (C57BL/6-background) were infected with a high dose of *T*. *muris* eggs. As stated previously, the mucin sulphation in the caecum of the naïve NaS1 KO mice was lower as compared to the wild-type mice [24]. This, however, did not have an effect on the establishment of infection (S4D Fig); similar number of worms were present, on day 12 pi., in the NaS1 KO and wild-type mice. Moreover, as infection progressed, the kinetics of worm expulsion were similar in the both wild-type and the NaS1 KO mice (S4D Fig).

### Recovery of sulphomucins in mice lacking the NaS1 transporter following *T*. *muris* infection

As we had demonstrated that the NaS1 KO mice expel *T*. *muris* over a similar time course as wild-type mice, RT-PCR was used to determine whether these mice mount a similar and concurrent T_H_2-type immune response to *T*. *muris* infection. The levels of *Il4* and *Il13* were elevated after infection in the NaS1 KO and wild-type mice compared to naïve mice (S5A and S5B Fig). A 2 to 6-fold increase in *Ifnγ* levels was also observed across the time course of infection (S5C Fig) in both NaS1 KO and wild-type mice although this did not significantly affect worm expulsion. The effect of the elevated IL-4/IL-13 levels was reflected in the histological analysis, as goblet cell hyperplasia. We have previously demonstrated that in the absence of the major intestinal mucin Muc2, results in a delay in the expulsion of the helminth *T*. *muris* from the host [2]. Importantly, *denovo* expression of the mucin Muc5ac in the niche of the helminth is critical for its expulsion [26]. However, in the NaS1 KO mice Muc2 and Muc5ac levels were similar compared to the WT mice (S5D–S5F Fig). HID-AB staining intensity showed that caecal mucin sulphation in the NaS1 knockout naïve mice was significantly less than in wild-type littermates (S4E and S4F Fig). However, surprisingly, as the infection advanced, the levels of mucin sulphation recovered in the NaS1 KO mice, and by day 25 of infection no difference was observed in the sulphation of the goblet cells in the caecal crypts (S4E and S4F Fig).

**Fig 4:**
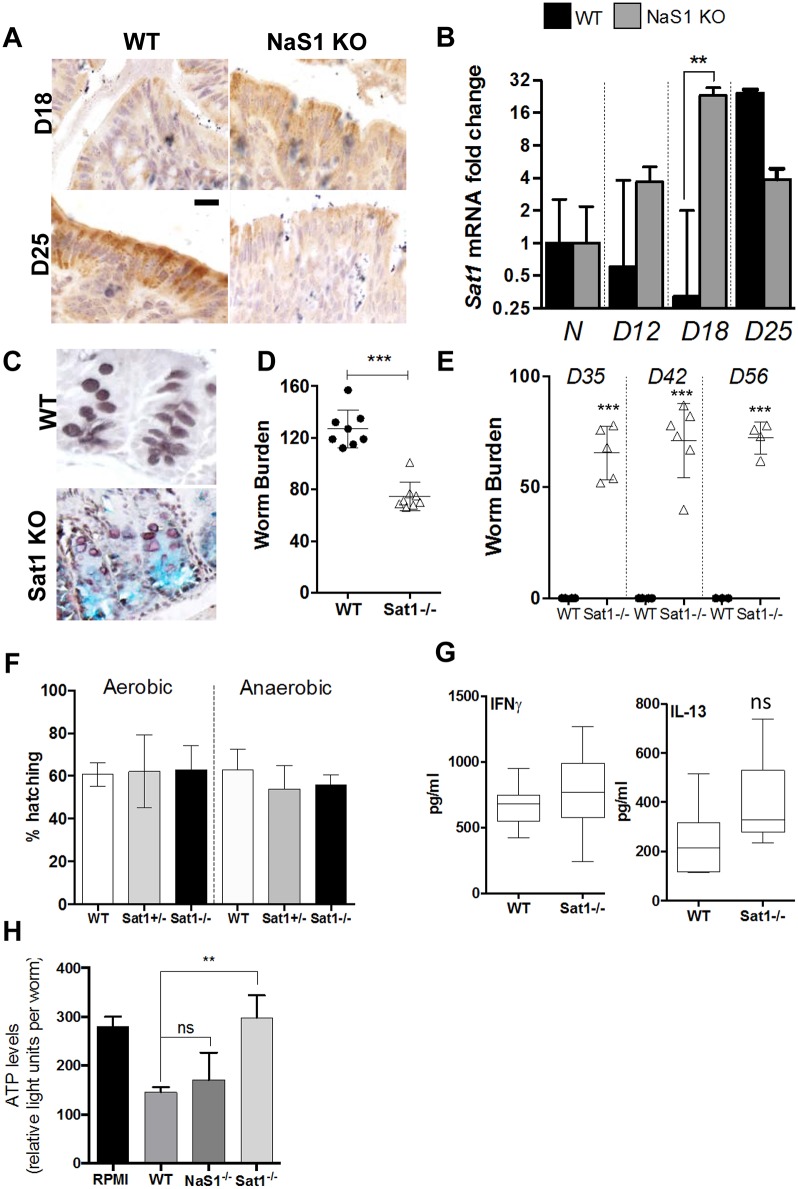
Epithelial sulphate transporter Sat1 promotes establishment and is important for the clearance of *T*. *muris* infection. Levels of Sat1 protein and mRNA were determined using (A) immunohistochemistry and (B) qRT-PCR, respectively, in WT and NaS1 KO mice during infection. (C) HID-AB staining illustrates the reduced mucin sulphation in naive Sat1 KO mice compared to WT mice. (D-E,G) Sat1 KO and WT mice were infected with 150 *T*. *muris* eggs. (D) Worm burden assessed on day 7 pi.; n = 8 mice per group. (E) Worm burden assessed on day 35, 42 and 56 pi. to confirm chronic infection. (F) Hatching of *T*. *muris* eggs determined after 24 h incubation at 37°C with mouse caecum from WT, Sat1 heterozygous mice and Sat1 KO mice under aerobic and anaerobic conditions. (G) ELISA was used to determine cytokine protein levels of IFNγ and IL-13 secreted by *T*. *muris* ESP–stimulated leukocytes isolated from mesenteric lymph nodes on day 7 pi. n = 5–6 mice per group. (H) Live worms were treated with RPMI medium only, or with caecal mucus isolated from uninfected WT, NaS1 KO or Sat1 KO mice (n = 12) for 24 h before measuring ATP levels. ATP levels are presented as relative light units per worm. Results are representative of the mean value of 100 worms per group ± SEM. One-way ANOVA with Bonferroni post-test. *P<0.05, **P<0.01, ***P<0.001 compared to WT mice. Scale bar = 100 μm.

As the *T*. *muris* infection progressed in the NaS1 KO mice, the depletion in the sulphomucin content within the goblet cells was reversed, suggesting that free sulphates, although lower systemically due to renal excretion, were transported efficiently through another sulphate transporter expressed in the caecal epithelium to be incorporated into the mucins. In addition to NaS1, sulphate anion transporter-1 (Sat1, Slc26a1) is the other major sulphate transporter in the caecum. Sat1 is expressed on the basolateral surface of epithelial cells and functions independently of NaS1, which is expressed on the apical membrane in the ileum, caecum and colon [32]. Therefore, we next assessed whether *Sat1* expression was altered in the NaS1 KO mice following infection. Using immunohistochemistry and RT-PCR, we observed a marked upregulation of *Sat1* mRNA in the NaS1 KO mice on day 18 pi., whereas, the up-regulation of *Sat1* occurred on day 25 pi. in the wildtype mice (Fig 4A and 4B). This suggested that Sat1 was compensating in the NaS1 KO mice during *T*. *muris* infection to improve sulphate uptake and availability as a substrate for incorporation into mucin oligosaccharides. In light of this, we sought to determine whether Sat1 KO mice also have reduced mucin sulphation in the caecum and whether this affects *T*. *muris* infection.

### Sat1 is essential for the establishment and expulsion of *T*. *muris* infection

HID-AB staining clearly showed that the mucins have low sulphation in Sat1 KO mice (Fig 4C). Interestingly, on day 7 pi., there was a significant reduction in the worm burden demonstrating a difference in the establishment of infection (Fig 4D). Despite lower establishment of *T*. *muris*, as infection progressed it was clear that the Sat1 KO mice were susceptible to chronic infection; these mice harboured infection until day 56 pi. and were unable to clear the infection (Fig 4E). The differences in establishment could be due to a variation in the efficiency of *T*. *muris* egg hatching. Therefore *T*. *muris* eggs were incubated *in-vitro* with caecal explants from WT mice and Sat1 heterozygous and KO mice overnight. However, there were no significant differences in the percentage of eggs hatched in WT or Sat1 KO mice under aerobic and anaerobic conditions (Fig 4F). Moreover, no differences in the immune response were observed at day 7 pi., where IFN-γ and IL-13 cytokine levels from cultured mesenteric lymph nodes were similar between WT and Sat1 KO mice (Fig 4G). We have previously shown that the mucins critical to worm expulsion adversely affect worm metabolism measured by ATP production [26], and therefore we analysed ATP production in worms exposed to sulphated and non-sulphated mucins. Sulphated mucins (isolated from the caecum of wild-type mice) lowered the energy levels of worms, whereas this effect was lost with decreased mucin sulphation (isolated from the caecum of Sat1 KO mice) directly implicating sulphated oligosaccharides in the anti-parasitic function of the mucins (Fig 4H). These findings in the Sat1 KO mice show that mucin sulphation is a critical element of effective helminth expulsion.

### Mucin glycosylation in the appendix changes during human *enterobius* infection

We investigated whether mucin sulphation and sialylation that are associated with chronic *T*. *muris* infection were also present in human helminthic infection. To this end in a pilot study we analysed specimens of vermiform appendix with histological evidence of *Enterobius vermicularis (EV)* infection and a control group of non- or mildly inflamed appendices with no histological evidence of infection (infection is unlikely but cannot be definitively excluded in this group). The cases were grouped into *EV* with or without mild non-erosive appendicitis (n = 10 and n = 29, respectively) and uninfected control appendices (n = 18). Infection with *EV* was associated with increased numbers of activated lymphoid follicles (*EV* vs control, mean 5.9 vs. 4.2, p = 0.05, Mann Whitney U t-test). Quantification of HID-AB staining illustrates that the intensity of mucin sulphation staining in *EV*-infected appendices was decreased compared to appendices lacking evidence of *EV* infection (S6 Fig).

## Discussion

**Fig 5:**
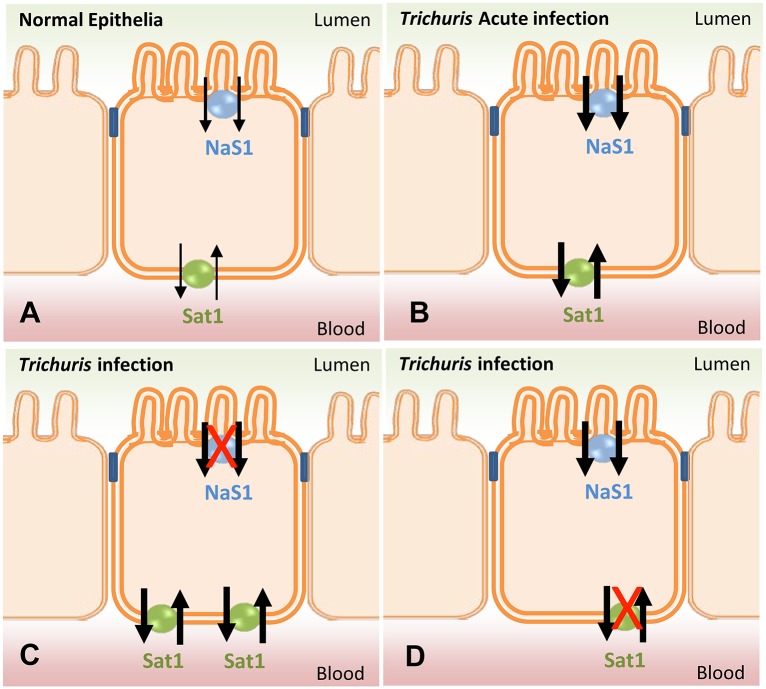
Schematic illustrating the important role of sulphate transporters during helminth infection. (A) In normal caecal epithelia, NaS1 expressed on the apical surface and Sat1 expressed on the basolateral surface is involved in the uptake of sulphates to maintain sulphate homeostasis and ensure sufficient substrate for sulphation of caecal mucins. (B) During acute *T*. *muris* infection, the demand for sulphation increases as a result of goblet cell hyperplasia, leading to the upregulation of NaS1 and Sat1, allowing uptake of sulphates from the luminal surface as well as blood, respectively. (C) In the absence of NaS1 blood sulphate decreases due to decreased intestinal absorption and renal reabsorption. Consequently, Sat1 on the basolateral membrane in the caecal epithelia is upregulated earlier in infection to ensure sufficient uptake of sulphates from blood. This compensation for the deficiency in blood sulphate leads to the recovery of sulphation on mucins ensuring effective worm expulsion. (D) In the absence of Sat1, mice develop hyposulfataemia and the cellular requirement for sulphates is unable to be met by NaS1, leading to reduced mucin sulphation. Reduced mucin sulphation reduces the efficiency of establishment of *T*. *muris* infection, but then prevents eradication of the infection.

The mucus barrier overlaying the intestinal epithelium is the first line of defence against enteric parasitic infections. We have previously demonstrated a protective role of mucins, the major component of the mucus barrier, in helminth expulsion [2]. It is thought that the glycans that decorate the mucin protein backbone, contribute to the protective properties of the mucus gel [1]. In this study, we demonstrate that sialylated mucins are mainly present in chronic helminthic infection. We described the changes in glycosylation in chronic and acute *T*. *muris* infection and show, in the strong T_H_2 environment that accompanies worm expulsion, that mucins were highly sulphated driven primarily by IL-13 mediated upregulation of goblet cell sulphotransferases. In contrast, predominantly sialylated mucins were found within the goblet cells during chronic *T*. *muris* infection in mice and in human appendices with *Enterobius* infection. Mice incapable of appropriate mucin sulphation could not expel *T*. *muris* demonstrating the importance of mucin sulphation as a key element of immune-driven worm expulsion. Providing a mechanistic explanation for the role of sulphated mucins in worm expulsion, we demonstrated that mucin polymers with high sulphomucin content were inherently more effective in repressing worm metabolism *in vitro* and were also more resistant to degradation by *T*. *muris* excretory secretory products. Using two genetic models of sulphate transporter deficiency we were able to establish the importance of mucin sulphation in both establishment of infection and subsequent immune clearance (see Fig 5).

Changes in mucin glycosylation have been reported to coincide with inflammation in several gastrointestinal diseases such as ulcerative colitis [33]. Moreover, alterations glycosylation of mucins have also been reported to occur during several models of helminth infection; *N*. *brasiliensis*, *T*. *spiralis* and *H*. *polgyrus* [10,11,34]. Whether these changes occur as a result of an on-going inflammatory response or as a result of an active change in order to resolve infection is not yet known. Our data suggests that distinct mechanisms regulate glycosyltransferases responsible for mucin sialylation and sulphation, corroborating previously published work [19] and definitively showing that IL-4/IL-13 receptor signalling preferentially drives expression of sulphotransferases. Histological analysis revealed that the changes in glycosylation with chronic *T*. *muris* infection were localised only within the caecum, consistent with the localised mucosal immune response. Interestingly, similar to goblet cell hyperplasia which is restricted to the caecum [2], no major changes in glycosylation were observed in the colon post *T*. *muris* infection. The changes in glycosylation were restricted to the helminths niche, it suggests these could be due to or a local immune response to/by the helminth itself. As human *EV* infection was also associated with an increased number of activated lymphoid follicles, this may represent an altered inflammatory milieu, which could contribute to the changes in mucin glycosylation. Mirroring the changes observed with the histology, an increase in the gene expression of sulphotransferases was observed in resistance, and sialotransferases were up-regulated in susceptibility. In chronic infection by *T*. *muris*, along with the down-regulation of goblet cell differentiation transcription factors, glycosylation within the goblet cells could possibly be perturbed. Indeed, there is evidence of a general loss of mature glycosylation on mucins in chronic infection [35]. The loss of sulphation was in naïve uninfected IL-4R KO mice when compared to the IL-4 KO mice, suggesting that in health the basal level of mucin sulphation is in part due to IL-13. In-vitro data showed that IL-13-treatment of colonic cell lines leads to an increase in sulphotransferase expression and subsequently protects the mucins from degradation by *T*. *muris* ESPs. Alterations in glycosylation could affect the hydration of the mucus gel and the viscosity of the barrier, which we have previously shown to be altered during worm expulsion [26]. It is important to acknowledge that in endemic areas, people are often infected with low doses of helminths, repeatedly. Therefore, the gradual change in glycosylation that occurs as a result of infection may eventually contribute to ‘acquired immunity’ and worm expulsion.

Parasitic excretory secretory products have the ability to degrade purified mucins [36]. The excretory secretory products (ESPs) of the *T*. *muris* helminth, that lives within the blanket of mucus, have been shown to be highly immunogenic [37]. However, whether ESPs affect crude mucus has not been previously investigated. Comparable amounts of mucus extracted from naïve NaS1 and wild-type mice were used to compare the effect of ESPs on low and highly sulphated mucins, respectively. After treatment with *T*. *muris* ESPs, the mucus with low sulphomucin content (from NaS1 KO mice) was reduced in size, which is evidence of degradation of the polymeric macromolecule responsible for the viscoelastic properties of the mucus and its ability to retain other host factors. This difference in the effect of ESPs was apparent on mucus with low sulphomucin content after only 2 hours of treatment, whereas ESPs had little effect on the mucus from wild-type naïve mice even after 6 hours. As we have demonstrated ESPs may be released as part of the helminths regime to promote its own survival, as the degradation of mucins would lead to diminishing the gel-like consistency of the mucus surrounding the worm and the delivery of anti-helminthic host factors [31]. Our findings here suggest that glycosylation plays an important role in preventing degradation, and that highly sulphated mucins protect the mucin protein core from the degradative effects of the helminths ESPs. Indeed studies of von Willebrand factor (vWF), which is a large multimeric glycoprotein homologous to gel-forming mucins, revealed that sialylated glycans increase susceptibility to its proteolysis [38].

We demonstrate that mucin sulphation patterns seen during *T*. *muris* infection influence both the establishment and expulsion of the parasite. Increased sulphation on intestinal mucins has been shown to reduce the establishment of the helminth *Strongyloides venezuelensis* [18]. We noted that depleted levels of sulphation using the Sat1 KO had a major effect on the establishment of *T*. *muris* infection. Of note, the caecum, which is the chosen niche of the *T*. *muris* helminth, is the highly sulphated. There is a possibility that sulphation is the cue for the *T*. *muris* eggs to hone in to the caecum as with reduced sulphation we did observe a decrease in establishment, no worms were found in the other parts of the intestine (small intestine, proximal colon or distal colon). The differences in establishment were not due to differences in the hatching of *T*. *muris* eggs. Together with our previous studies [2,26] we now know that mucins are critical to expulsion, MUC5AC is more important than MUC2, oligomerisation of the mucins is not required for the adverse effects on metabolism, and sulphated oligosaccharides are required. How the long intact sulphated mucin molecules transmit their adverse effects on worm metabolism remain to be demonstrated. No effect on *T*. *muris* establishment was noted in the NaS1 KO mice, possibly because the depletion in sulphation was not sufficient to have an effect on worm establishment. In the absence of the apical sulphate transporter, NaS1, these hyposulphataemic mice up-regulated the expression of Sat1 transporter, which is involved in the uptake of sulphates from blood [32]. Interestingly, Sat1 is elevated with the increasing demand for sulphates during the rejection of *T*. *muris*, earlier in the NaS1 KO mice compared to the wild-type mice. Sat1 levels return almost to baseline in the NaS1 KO mice by day 25 pi., which is when the goblet cell hyperplasia seemed to subside. Such evidence suggests that Sat1 expression is up-regulated in response to the increased cellular requirement of sulphates, rather than the immune response and is effectively scavenging sufficient sulphate to effectively sulphate the mucins and ensure worm clearance. Importantly, the sustained sulphation observed in the acute *T*. *muris* model is essential for the expulsion of the helminth from the host.

In summary, we have demonstrated that maintained mucin sulphation, influenced by the T_H_2 type immune response, is clearly a feature of resistance to *T*. *muris* infection. This has likely evolved as a part of the immune response against *T*. *muris* as increased sulphation changes the properties of the mucus barrier making the mucins more resistant to degradation. Depleted levels of mucin sulphation, have a significant effect on the establishment of the *T*. *muris* infection that appear independent of changes in the microbiome. With the high demand of cellular sulphate during *T*. *muris* infection, Sat1 was up-regulated in the caecal epithelium and is essential for efficient mucin sulphation with deficiency leading to inability to clear infection. Given the essential role of mucins in clearing helminth infections, this study highlights the complex process by which alterations in mucin glycosylation occur following infection and contribute to the establishment and clearance of infection.

## Materials and methods

### Animals

AKR, BALB/c (Harlan Olac), IL-4 KO and IL-4R KO (BALB/c-background) mice were maintained in the Biological Services Unit at the University of Manchester. All mice used were at 6–10 week old male mice. The protocols employed were in accordance with guidelines by the Home Office Scientific Procedures Act (1986). NaS1 knockout (KO) and Sat1 KO mice (4–12 weeks old male C57BL/6) and their wild-type littermates originally produced by gene mutation [24,32] were housed at the Mater Medical Research Institute and experiments were approved by the University of Queensland Animal Experimentation Ethics Committee. All mice were kept in sterilized, filter-topped cages, and fed autoclaved food.

### Human *enterobius* infection

Cases of *enterobius vermicularis* infestation of the vermiform appendix over the period 2011–2014 were identified in the formalin fixed paraffin embedded archive of Mater Pathology Services. All cases were reviewed by an anatomical pathologist (RL) and anatomical pathology trainee (PH) to confirm the diagnosis. Cases with more than mild lamina propria acute inflammation were discarded to exclude confounding effects from other sources of inflammation. The cases were grouped into *EV* with or without mild non-erosive appendicitis (n = 10 and n = 29, respectively) and uninfected control appendices (n = 18). 5μm thick sections were cut onto Superfrost coated slides (Thermo Scientific, Braunschweig) and submitted for staining as per below. Use of human specimens was approved by the Mater Health Services Human Research Ethics Committee (reference 15MHS69) and Research Governance Office (reference RG-15-147).

### Parasitological technique

The techniques used for *T*. *muris* maintenance and infection were described previously [39]. Mice were orally infected with approximately 100–300 eggs for a high dose infection and >15 eggs for a low dose infection. Worm burdens were assessed by counting the number of worms present in the caecum as described previously [39]. For ATP measurements, live worms were extracted from the caecum of Rag1^-/-^ mice, which was longitudinally cut and segmented before incubation with 0.1M NaCl for 2 hours at 37◦C with frequent shaking. Worms were counted after separation from debris and epithelial cells using a 0.7μm filter and kept in RPMI-1640 supplemented with 10% FCS. Live worms were incubated with either RPMI or mucus isolated from 6 wk old WT, NaS1 KO or Sat1 KO mice for 48 h. Alive worms were subsequently homogenised. The CellTiter-Glo luminescent cell viability assay was carried out according to manufacturer’s instructions (Promega Corp., USA). Relative light units (RLUs) were calculated per worm: *RLU* = (sample light units − blank light units)/number of worms. Substrate only was used as a blank control.

### Histology, immunohistochemistry and immunofluorescence microscopy

A 1cm segment or the whole tissue (rolled) was fixed in 10% neutral buffered formalin or 95% ethanol and processed using standard histological techniques. Sections were treated with 0.1M potassium hydroxide for 30 minutes prior to staining with periodic acid Schiff’s reagent (PAS). Slides were counterstained with either haematoxylin and eosin or 1% fast-green. To assess mucin sulphation, sections were stained with High Iron Diamine-Alcian Blue (HID-AB) as previously described [40]. Standard immunohistochemical staining methods [41] were used for immunohistochemistry with monoclonal Sat1 (Sulphate anion transporter 1) antibody [32].

### RT-PCR

RNA from epithelial cells was isolated using the previously described method [42]. cDNA was generated using an IMPROM-RT kit (Promega) or Superscript III (Invitrogen). Absolute QPCR SYBR Green (ABgene) was used for quantitative PCR. Primer efficiencies was determined using cDNA dilutions and genes of interest (S1 Table) were normalised against the housekeeping gene, β-actin, and expressed as a fold difference to uninfected naïve message levels. RT-PCR products were directly sequenced to verify the identity of the amplified genes. In brief, products were digested with Exonuclease I and Calf Intestinal Phosphatase and subsequently sequenced using the ABIPRISM Big-Dye Terminator cycle sequencing reaction at the Sequencing Facility in the University of Manchester. The data was analysed using Chromas Pro v1.34 and the sequences obtained were compared against the GenBank database (http://www.ncbi.nlm.nih.gov/BLAST).

### In-vitro culture

LS174T cells (originally obtained from ATCC) were cultured as previously described [31]. In brief, cells were cultured in high glucose DMEM with 2mM L-glutamine, 100 U/mL penicillin, 100 g/mL streptomycin and 10% FBS until confluent (70–80%). Cells were then treated with 50 ng/mL of recombinant human IL-13 or IFNγ for 24 h and samples were taken for protein/RNA or rate zonal centrifugation. For hatching experiments, sections of mouse caeca or caecal extracts were isolated and kept overnight with *T*. *muris* eggs at 37°C and 5% CO_2_ or at 10% CO_2_, 10% H_2_, 80% N_2_.

### Mucus extraction and analysis by agarose gel electrophoresis

Caeca from NAS1 KO mice and their wild-type littermates were gently flushed with PBS to remove the faecal matter. The mucus was lightly scraped and lyophilized and subsequently solubilised in 6M urea, reduced using 50mM dithiothreitol (DTT) and carboxylmethylated using 0.125M iodoacetamide. Samples were electrophoresed on 1% (w/v) agarose gels in TAE buffer (40mM Tris acetate and 1mM EDTA, pH8) and 0.1% SDS at 30 volts for 15 hours. After electrophoresis, mucins were transferred to a nitrocellulose membrane by vacuum blotting in 0.6M sodium chloride and 60mM sodium citrate at a pressure of 45–50 mbar for 2 hours detected using PAS, HID staining or probed with the MUC2 antibody [43].

### Treatment with *T*. *muris* excretory secretory products

The excretory secretory products (ESPs) were collected using methods previously described [44]. Aliquots of crude mucus scrapes (in equal volumes of PBS) were incubated at 37°C with the ESPs at 50 μg/ml for various time points (as specified). Control samples were not treated with the ESPs, but were incubated at 37°C for the maximum time point as previously described [31].

### Cytokine measurements from cultured mesenteric lymph nodes

Mesenteric lymph nodels (mLNs) were removed, cells were isolated and resuspended at 5 x 10^6^ cells/mL in RPMI 1640 with 10% FBS, 2 mM L-glutamine, 100 U/mL penicillin, 100 μg/mL Streptomycin. Cultures were stimulated with 50 μg/mL of ESPs for 24 h at 30°C and 5% CO_2_. Cell free supernatants were stored at -80°C. IFNγ and IL-13 levels were determined using ELISAs as per manufacturer’s instructions (R&D).

### Rate zonal centrifugation

6–8 M guanidinium chloride gradients were formed in centrifuge tubes using an MSE gradient maker connected to a Gilson Minipuls 2 peristaltic pump. Mucin samples (in 4 M guanidinium chloride) were loaded onto the gradient and centrifuged in a Beckman Optima L-90K Ultracentrifuge using a Beckman SW40 rotor at 40,000 rpm for 2.45 hours at 15°C. Fractions were taken from the top of the tubes, analysed by slot blotting and PAS-staining [45]. The refractive index of each fraction was measured using a refractometer to determine the guanidinium chloride concentration; the gradients were comparable.

### Quantification of histological staining

All histological analysis was done blinded. Sulphomucin containing crypts within the caecum (identified as blackish goblet cells) were quantified and compared to the total number of crypts. The numbers of goblet cells expressed per crypt were counted in 20–50 longitudinally sectioned crypt units. The area stained (pixel/mm^2^) per 20–50 crypts was determined by using the ImageJ software version 1.39a. The goblet cell staining intensity was measured using the BioRad GS-800 densitometer in 250 goblet cells.

### Statistical analysis

All results are expressed as the mean ± SEM. Statistical analysis was performed using SPSS version 16.0 or GraphPad Prism version 6.0e. Statistical significance of different groups was assessed by using non-parametric tests (all figure legends describe the analysis used). P<0.05 was considered statistically significant.

## Supporting information

S1 Fig (A) BALB/c or AKR mice were infected with high dose (HD) of *T*. *muris* eggs (150) or a low dose (~12). Worm burdens were assessed on day 14, 21 and 35 post infection. Note same set of BALB/c naïve controls were used for both acute and chronic infection BALB/c models. *P<0.05, **P<0.01, ***P<0.001 compared to naïve mice. One-way ANOVA with Bonferroni post-test. (B) HID-AB staining of the colon of all three infection models. Scale bar = 100μm.
(PNG)

S2 Fig qRT-PCR of caecal tissue for *Ifng* (A), *Il13* (B) and *Muc2* (C) from BALB/c mice infected with a low dose (chronic infection) or a high dose (acute infection) of *T*. *muris* eggs (corresponding data shown in Figs 1 and 2). Red dashed line = naïve levels. Results represent the mean value of 5–7 mice per group ± SEM. *P<0.05, **P<0.01, ***P<0.001 compared to naïve mice. One-way ANOVA with Bonferroni post-test.
(PNG)

S3 Fig (A) Worm burdens were assessed in wild-type (WT), IL-4 knockout (KO) and IL-4R KO mice on day 18 and 32 post infection with 150 *T*. *muris* eggs. (B) qRT-PCR was used to determine the mRNA levels of transferases (*Gal3ST1*, *GlcNAcST2*, *GlcNAC6ST3*) in the caecal mucosa of WT, IL4 KO and IL4R KO mice. (C) LS174T cells were treated with PBS (control) or 50 ng/mL of recombinant human IL-13 for 24 h, cell lysates were collected and analysed using agarose gel electrophoresis and western blotting for MUC2 (n = 4). LS174T cells were treated with (D) IL-13 or (E) IFNγ and qRT-PCR was used to determine the changes in sulphotransferases (*Gal3St1*, *GlcNAcST2*) and sialyltransferases (*ST3Gal*, *ST8GalNAc*). N = 8. (F) Control, IL-13-treated or IFNγ-treated LS174T cell mucins were treated with *T*. *muris* ESPs for 6 h, extracted and subjected to rate zonal centrifugation. Fractions were transferred to nitrocellulose membrane, stained with PAS and staining intensity was measured. Results are presented as the mean value of n = 8 per condition. (G) qRT-PCR was used to determine the mRNA levels of sulphotransferases in the caecal mucosa of WT and IL4R KO mice. *P<0.05, **P<0.01, ***P<0.001 compared to controls, Mann-Whitney U non-parametric t-test.
(PNG)

S4 Fig (A) HID-AB staining of caecal tissue from WT and NaS1 KO mice to assess the level of sulphation. (B) Caecal mucus from WT and NaS1 KO mice analysed by agarose gel electrophoresis and stained with HID-AB (sulphated mucins) or PAS staining (total glycoproteins levels); presented as the percentage of HID-AB staining intensity relative to total PAS. N = 4. (C) Crude mucus from WT and NaS1 KO mice was untreated or treated for 2 or 6 h with 50 μg/mL of *T*. *muris* ESPs, then extracted and subjected to rate-zonal centrifugation. Fractions were transferred to nitrocellulose membrane, stained with PAS and staining intensity measured. Results are presented as the mean value of 3–5 mice per group. 6h WT and NaS1 KO mucus treated with ES for 6 h is presented as a percentage of area under the curve (AUC) of fractions (Fr) 1–9, 10–18 and 19–24 from untreated (−ES) and ESP-treated (+ES) mucus isolated from 5–7 WT and NaS1 KO mice. (D-F) WT and NaS1 KO mice were infected with ~300 *T*. *muris* eggs. (D) Worm burdens assessed on day 12, 18 and 25 pi. (E) Quantitation of HID staining intensity per 250 goblet cells and (F) representative examples of HID-AB staining illustrating the changes in glycosylation during infection. Results represent the mean ± SEM of 5–7 mice per group. One-way ANOVA with Bonferroni post-test. ***P<0.001 compared to WT mice. Scale bar = 100 μm.
(PNG)

S5 Fig qRT-PCR was used to determine the levels of T_H_2 cytokines (A) *Il4*, (B) *Il13* and T_H_1 cytokine (C) *Ifng* during infection in the WT and NaS1 KO mice. (D) Number of goblet cells were counted per crypt in the caecum during infection using PAS staining (E). qRT-PCR was used to assess the changes in (F) Muc2 and (G) Muc5ac mRNA during infection in WT and NaS1 KO mice. N = 5–7 mice per group.
(PNG)

S6 Fig (A) HID-AB staining intensity was quantified in control and *Enterobius vermicularis* (*EV*) infected samples. 4 fields of view per sample were used to determine the mean per sample; data is presented as intensity of staining per 250 goblet cells. Representative micrographs from n = 39 EV infected and n = 18 uninfected control appendices are shown. ***P<0.001, Mann Whitney-U t-test.
(PNG)

S1 Table Forward and reverse primer sequences of the genes of interest.
(TIF)
