# Supplementary figures and images for: Immune-driven alterations in mucin sulphation is an important mediator of Trichuris muris helminth expulsion
Source: PLoS Pathog. 2017 Feb 13;13(2):e1006218. doi: 10.1371/journal.ppat.1006218 (PMC5325613; doi:10.1371/journal.ppat.1006218)

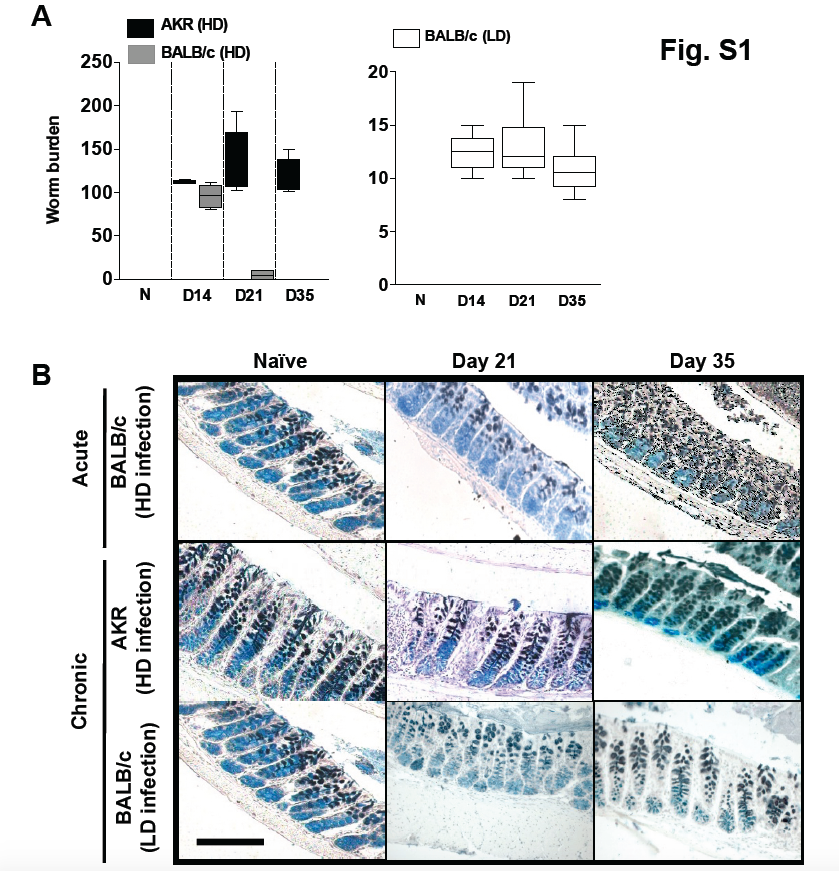

Supplement: S1 Fig — (A) BALB/c or AKR mice were infected with high dose (HD) of T. muris eggs (150) or a low dose (~12). Worm burdens were assessed on day 14, 21 and 35 post infection. Note same set of BALB/c naïve controls were used for both acute and chronic infection BALB/c models. *P<0.05, **P<0.01, ***P<0.001 compared to naïve mice. One-way ANOVA with Bonferroni post-test. (B) HID-AB staining of the colon of all three infection models. Scale bar = 100μm. (PNG) [file ppat.1006218.s001.png]

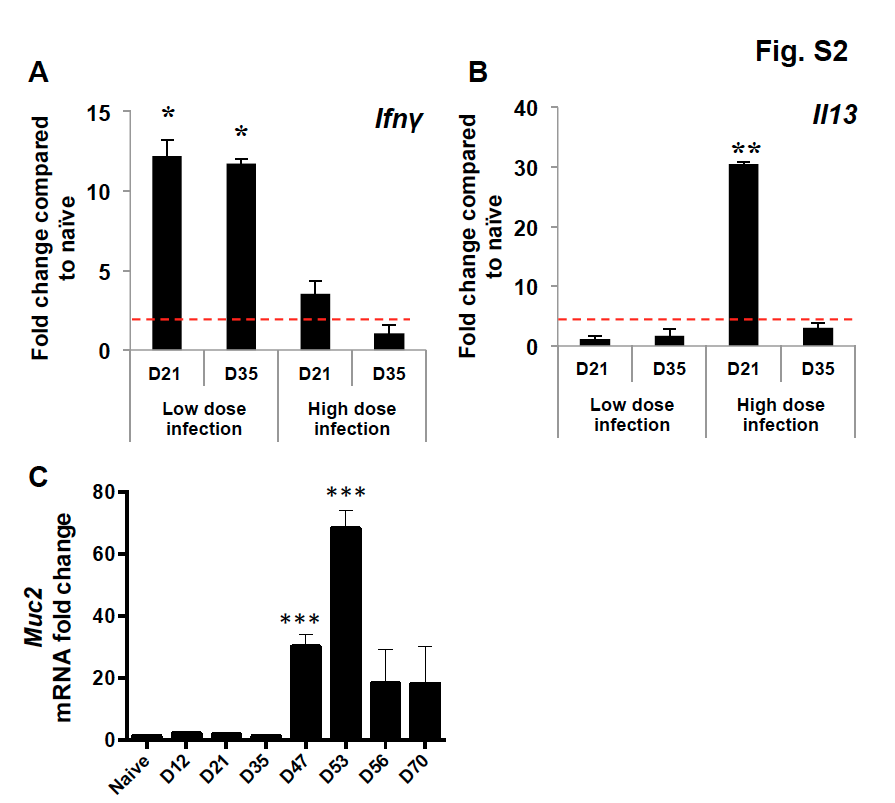

Supplement: S2 Fig — qRT-PCR of caecal tissue for Ifng (A), Il13 (B) and Muc2 (C) from BALB/c mice infected with a low dose (chronic infection) or a high dose (acute infection) of T. muris eggs (corresponding data shown in Figs 1 and 2). Red dashed line = naïve levels. Results represent the mean value of 5–7 mice per group ± SEM. *P<0.05, **P<0.01, ***P<0.001 compared to naïve mice. One-way ANOVA with Bonferroni post-test. (PNG) [file ppat.1006218.s002.png]

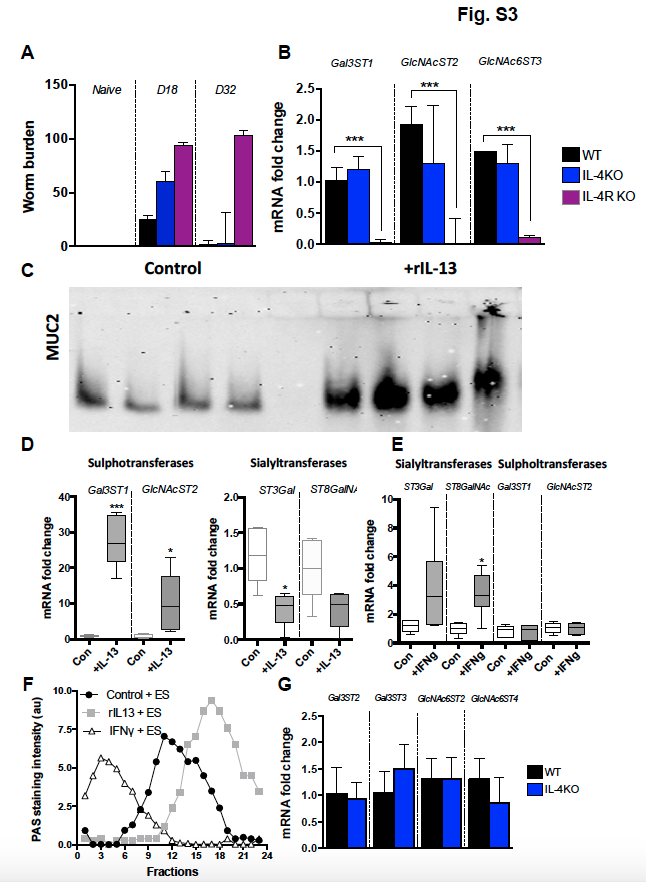

Supplement: S3 Fig — (A) Worm burdens were assessed in wild-type (WT), IL-4 knockout (KO) and IL-4R KO mice on day 18 and 32 post infection with 150 T. muris eggs. (B) qRT-PCR was used to determine the mRNA levels of transferases (Gal3ST1, GlcNAcST2, GlcNAC6ST3) in the caecal mucosa of WT, IL4 KO and IL4R KO mice. (C) LS174T cells were treated with PBS (control) or 50 ng/mL of recombinant human IL-13 for 24 h, cell lysates were collected and analysed using agarose gel electrophoresis and western blotting for MUC2 (n = 4). LS174T cells were treated with (D) IL-13 or (E) IFNγ and qRT-PCR was used to determine the changes in sulphotransferases (Gal3St1, GlcNAcST2) and sialyltransferases (ST3Gal, ST8GalNAc). N = 8. (F) Control, IL-13-treated or IFNγ-treated LS174T cell mucins were treated with T. muris ESPs for 6 h, extracted and subjected to rate zonal centrifugation. Fractions were transferred to nitrocellulose membrane, stained with PAS and staining intensity was measured. Results are presented as the mean value of n = 8 per condition. (G) qRT-PCR was used to determine the mRNA levels of sulphotransferases in the caecal mucosa of WT and IL4R KO mice. *P<0.05, **P<0.01, ***P<0.001 compared to controls, Mann-Whitney U non-parametric t-test. (PNG) [file ppat.1006218.s003.png]

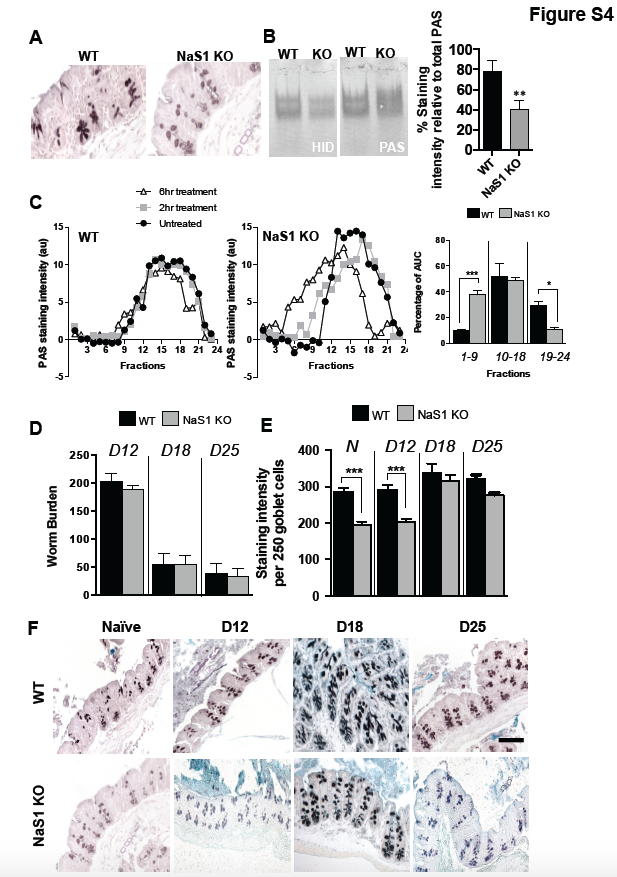

Supplement: S4 Fig — (A) HID-AB staining of caecal tissue from WT and NaS1 KO mice to assess the level of sulphation. (B) Caecal mucus from WT and NaS1 KO mice analysed by agarose gel electrophoresis and stained with HID-AB (sulphated mucins) or PAS staining (total glycoproteins levels); presented as the percentage of HID-AB staining intensity relative to total PAS. N = 4. (C) Crude mucus from WT and NaS1 KO mice was untreated or treated for 2 or 6 h with 50 μg/mL of T. muris ESPs, then extracted and subjected to rate-zonal centrifugation. Fractions were transferred to nitrocellulose membrane, stained with PAS and staining intensity measured. Results are presented as the mean value of 3–5 mice per group. 6h WT and NaS1 KO mucus treated with ES for 6 h is presented as a percentage of area under the curve (AUC) of fractions (Fr) 1–9, 10–18 and 19–24 from untreated (−ES) and ESP-treated (+ES) mucus isolated from 5–7 WT and NaS1 KO mice. (D-F) WT and NaS1 KO mice were infected with ~300 T. muris eggs. (D) Worm burdens assessed on day 12, 18 and 25 pi. (E) Quantitation of HID staining intensity per 250 goblet cells and (F) representative examples of HID-AB staining illustrating the changes in glycosylation during infection. Results represent the mean ± SEM of 5–7 mice per group. One-way ANOVA with Bonferroni post-test. ***P<0.001 compared to WT mice. Scale bar = 100 μm. (PNG) [file ppat.1006218.s004.png]

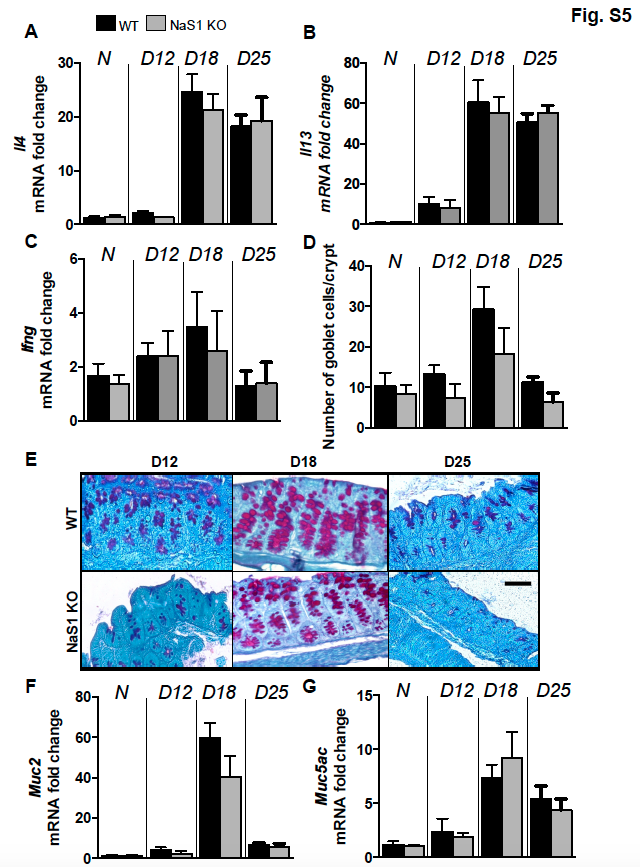

Supplement: S5 Fig — qRT-PCR was used to determine the levels of TH2 cytokines (A) Il4, (B) Il13 and TH1 cytokine (C) Ifng during infection in the WT and NaS1 KO mice. (D) Number of goblet cells were counted per crypt in the caecum during infection using PAS staining (E). qRT-PCR was used to assess the changes in (F) Muc2 and (G) Muc5ac mRNA during infection in WT and NaS1 KO mice. N = 5–7 mice per group. (PNG) [file ppat.1006218.s005.png]

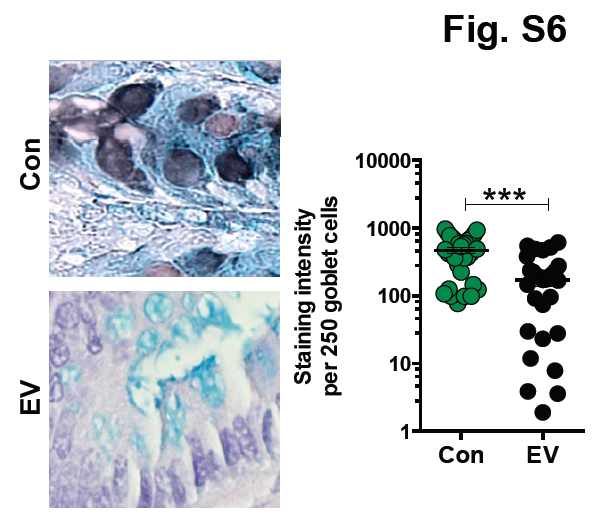

Supplement: S6 Fig — (A) HID-AB staining intensity was quantified in control and Enterobius vermicularis (EV) infected samples. 4 fields of view per sample were used to determine the mean per sample; data is presented as intensity of staining per 250 goblet cells. Representative micrographs from n = 39 EV infected and n = 18 uninfected control appendices are shown. ***P<0.001, Mann Whitney-U t-test. (PNG) [file ppat.1006218.s006.png]
